# Rectal Feeding

**Published:** 1906-02-17

**Authors:** 


### RECTAL FEEDING.

Dr. F. D. Boyd communicated to the Medico-Chirurgical Society of Edinburgh on February 7 the results of a clinical research on the value of rectal feeding. The patients, six in number, were all the subjects of gastric ulcer, and Dr. Boyd was able to investigate the metabolism in these six cases by means of accurate estimations. The absorption of proteid as tested by the injection of pancreatised egg-albumen was found to be extremely small, and bore no relation to the amount injected, leading to the conclusion that the use of white of egg in nutrient enemata is a very deceptive and extravagant proceeding. Fats in the form of yolk of egg are much more largely absorbed, while carbohydrates in the form of pure dextrose were taken up by the bowel in considerable quantities, and in a fairly constant proportion to the amount contained in the enema. No irritant effects had been observed after the injection of even large amounts of sugar, those noted by other observers being perhaps due to the use of inferior dextrose, which is liable to contain sulphuric acid. It having been stated that sugars are liable to be decomposed in the bowel, and are therefore of little value as enemata, experiments were made to determine the extent of such loss, which in four hours was found to be less than 1 per cent. The total value of the absorption was never in the most favourable cases more than 500 calories per diem, or less than a third of what is required for the balance of nutrition, so that rectal feeding was subnutrition of a pronounced type. Dr. Boyd suggests that nutrient enemata should be made up to ten ounces instead of three or four ounces, as is usual at present, and should contain milk and dextrose instead of proteids. This amount is easily retained if given by a syphon funnel, and does not require repetition oftener than every six hours.

